# Neonatal Outcomes in Labor After Intravenous Remifentanil Analgesia vs. Epidural Analgesia: A Retrospective Observational Study

**DOI:** 10.7759/cureus.56327

**Published:** 2024-03-17

**Authors:** Suzana Sobot Novakovic, Sanja Cuk, Dragan Rakanovic, Dragana Loncar Stojiljkovic, Branka Cancarevic Djajic, Miroslav Gajic

**Affiliations:** 1 Anesthesiology and Critical Care, University Clinical Centre of the Republic of Srpska, Banja Luka, BIH; 2 Anesthesia and Critical Care, University Clinical Centre of the Republic of Srpska, Banja Luka, BIH; 3 Anesthesiology and Critical Care, Faculty of Medicine of the University of Banja Luka, Banja Luka, BIH; 4 Obstetrics and Gynaecology, University Clinical Centre of the Republic of Srpska, Banja Luka, BIH; 5 Neonatology, University Clinical Centre of the Republic of Srpska, Banja Luka, BIH

**Keywords:** labor and birth, neonatal outcome, remifentanil, labor epidural analgesia, labor pain managment

## Abstract

Background

Some evidence indicates that maternal analgesia during labor may have adverse effects on neonates due to exposure to specific drugs or the potential effects of analgesia on the course of labor. We assessed the clinical outcome of term neonates born to mothers who received epidural analgesia (E) or systemic analgesia with remifentanil (R) during labor.

Methods

Data was collected retrospectively over one year. We have evaluated the medical records of 247 full-term neonates; 208 were born to mothers who received E and 39 to mothers who received R. Data on Apgar scores and neonatal complications (perinatal asphyxia, respiratory distress, infection, hyperbilirubinemia, and birth injuries), and average hospital stay were collected. Mann-Whitney U test, chi-square test, and logistic regression analysis were used where appropriate.

Results

The values of the mean Apgar scores between E and R at 1 and 5 minutes were similar (8.83 vs. 8.97, *p* = 0.252; 9.81 vs. 9.87, *p* = 0.762, respectively). The average length of neonatal hospitalization did not differ between groups (4.19 vs. 4; *p* = 0.557). The percentages of neonates with any complication were similar between groups (28.3% vs. 32.5%, *p* = 0.598). Neonates born by cesarean delivery (CD) had significantly worse outcomes than those born vaginally (*p* = 0.008, OR 2.8, 95% CI [1.30, 6.17]).

Conclusion

We did not find a statistically significant difference in mean Apgar scores and neonatal complications between neonates who received epidural vs. remifentanil analgesia. An increased rate of complications in neonates born via CD was found. Future studies should have a larger sample size and be powered to detect associations in these findings.

## Introduction

Pain management during labor is one of the most important factors for patient satisfaction. Epidural analgesia (EA) represents the most efficient and widely accepted pain relief method during labor, with a rate of use between 20% and 80% of all deliveries [1]. There are also other pain management options, like the use of opioids. Remifentanil is probably the most popular among opioids because of its short-acting characteristics [2]. It serves as an alternative to epidural anesthesia when an epidural is not feasible or desired. Research indicates that remifentanil provides better pain relief than pethidine but is less effective than an epidural [3].

Safe childbirth and a positive outcome for mothers and neonates during labor are imperative for every medical professional and health facility. That is why any side effects from pain management used during labor are observed. Medical professionals always try to outweigh the risks that come with the procedure. Studies have previously demonstrated an increased rate of the prolonged second stage of labor, instrumental delivery (ID), and maternal fever with EA [4,5]. All of these factors can potentially increase neonatal morbidity. Labor analgesia with intravenous remifentanil has been reported to be associated with adverse effects, including excessive maternal sedation and oxygen desaturation, as well as fetal heart rate decelerations, depending on the dose and administration regimen [6]. Neonates theoretically could experience the same adverse effects as mothers. However, there are no case reports/studies of serious neonatal adverse events with maternal remifentanil use, although most of the studies tested neonatal effects as their secondary outcomes [7].

Most of the studies regarding pain management in labor investigated the effects on the mother and the course of labor [4,8]. Fewer studies examined the effects on neonates, and most of those reported neonatal effects as a secondary outcome in the immediate postnatal period (in the first 24 hours after birth) [9].

This study aimed to compare the effects of epidural analgesia (EA) and remifentanil analgesia (RA) during labor on neonatal outcomes in the immediate (first 24 hours) and early postnatal periods (from day two until discharge from the hospital). The primary objective was to compare Apgar scores and immediate complications in the first 24 hours after birth. Secondary objectives were to analyze early postnatal complications, 24 hours after birth until discharge from the hospital, and obstetric outcomes.

We hypothesize that RA has less impact on the course of labor and, thus, better neonatal outcomes compared to EA. 

## Materials and methods

The study was submitted for approval by the Ethics Committee of the University Clinical Centre of the Republic of Srpska (UCC RS) on February 9, 2017, and received certificate No. 01-9-21.2/17. This was a retrospective, observational study. We searched records from the patients who gave birth with EA or RA from the UCC RS hospital electronic patient record system from January 2017 until December 2017. All the detailed clinical maternal and neonatal information, including delivery mode and demographic data such as age, gestational characteristics, complications during delivery, and previous medical and obstetrical history, were documented and analyzed.

Inclusion criteria were vaginal delivery with EA or RA, patients 18 years of age or older, and healthy-term singleton pregnancy. Exclusion criteria included multiple pregnancies, non-cephalic presentation, preeclampsia, low birth weight in neonates, incomplete electronic record data.

According to protocols in our hospital, EA was performed by placing epidural catheters in L3-4 and L4-5 intervertebral epidural spaces. An anesthesiologist administered EA according to the patient's demand with cervical dilatation of more than 3 cm. Analgesia was maintained with a 0.1% levobupivacaine solution with 2 mcg/ml fentanyl (bolus, continuous infusion, or PCA). RA was administered by the anesthesiologist on the patient's demand and was maintained with a 50 mcg/ml solution of opioid, starting with a bolus of 0.5 mcg/kg and continuous infusion afterward in doses of 0.05-0.2 mcg/kg/min.

IBM Corp. Released 2011. IBM SPSS Statistics for Windows, Version 20.0. Armonk, NY: IBM Corp. was used for data processing. The results are presented using tables. A t-test was utilized for comparing continuous variables. The analysis of categorical variables was performed using Fisher's exact test and the chi-square test. Multivariable logistic regression analyses were used. The skewness and kurtosis test were conducted to deal with the normality issue, and depending on the results, the appropriate test has been applied. In normally distributed variables, the Mann-Whitney test has been applied. A p-value of <.05 was considered statistically significant for all analyses.

The primary objective was to compare Apgar scores and immediate neonatal complications such as perinatal asphyxia and respiratory distress between groups in the first 24 hours after delivery. The secondary objective was to detect early neonatal complications after 24 hours of life until discharge from the hospital, such as infection, hyperbilirubinemia, birth injuries, admission to the neonatal intensive care unit (NICU), special care baby unit (SCBU), and prolonged hospital stay.

## Results

During the 12 months, we identified medical records of 256 full-term neonates from mothers who received analgesia during labor. Out of these, 247 met the inclusion criteria: 208 in the epidural group (group E) and 39 in the remifentanil group (group R). Demographic data on mothers and neonates is presented in Tables 1, 2. No differences were recorded between groups, except that significantly more patients received EA compared to RA during labor. 

**Table 1:** Patients' characteristics and chronic diseases Data are presented as means, numbers, or percentages. E: epidural group; R: remifentanil group. The significance threshold was set at p < 0.05. *Chronic diseases detected in patients were thrombophilia, hypertension in pregnancy, heart defects, hyper- or hypothyreosis, and thrombocytopenia.

Group	E (N= 208)	R (N=39)	p-value
Age (mean±SD)	30.8 ± 5	32.7 ± 5	0.135
Gestation (mean±SD)	39.4 ± 1	39.5 ± 2	0.363
Weight (kg) (mean±SD)	81±13	84±14	0.147
BMI (kg/m^2^) (mean±SD)	29.1 ± 4.7	29.8 ± 5.4	0.519
Chronic diseases* N (%)	31 (14.9)	8 (20.51)	0.378

**Table 2:** Neonatal characteristics Mean values are utilized for presenting the data. The significance threshold was set at p < 0.05.

Group	E (N=208) mean ± SD	R (N=39) mean ± SD	p-value
Birth weight (g)	3563.3 ± 423.3	3506.9 ± 396.6	0.362
Birth length (cm)	52.8 ± 1.79	52.5 ± 1.40	0.373
Apgar 1 min	8.83 ± 0.79	8.97 ± 0.52	0.252
Apgar 5 min	9.81 ± 0.16	9.87 ± 0.33	0.762

No differences were observed between the groups regarding the duration of labor and type of delivery. Indications for CD included fetal distress, prolonged labor, and cephalopelvic disproportion. None of these indications showed significant differences between the groups, as shown in Table 3.

**Table 3:** Obstetric data Data are presented as means, numbers, and percentages. The significance threshold was set at p < 0.05. NC: not able to calculate. *Complications during vaginal delivery that were indications for CD.

Group	E (N=208)	R (N=39)	p-value
Primiparous N (%)	135 (64.9)	29 (74.3)	0.251
Multiparous N (%)	73 (35.1)	10 (25.6)	0.251
Analgesia duration (mean ± SD)	359.5 ± 221.2	317.1 ± 179.7	0.375
Spontaneous labor N (%)	181 (86.2)	35 (89.8)	0.535
Instrumental delivery	0	0	NC
Cesarean section N (%)	29 (13.8)	4 (10.2)	0.535
Fetal distress* N (%)	9 (4.33)	3 (7.69)	0.285
Prolonged labor* N (%)	11 (5.29)	1 (2.56)	0.111
Cephalopelvic disproportion* N (%)	8 (3.85)	0	NC

In both groups, neonates experienced complications, with an incidence of around 30%. The most frequent complication was infection, with an incidence of 20% in both groups. The main criteria for diagnosing infection in all neonates was elevated C-reactive protein above 10 mg/L. Hyperbilirubinemia was the second most common complication, with an incidence of 5% in both groups. Only five neonates were admitted to the NICU, all of them from the epidural group. The reasons for NICU admission were perinatal asphyxia and respiratory distress. The cause of perinatal asphyxia in two neonates was intracerebral hemorrhage. The number of neonates admitted to the SCBU in the E group was higher compared to the R group, but without statistical significance. Reasons for admission to SCBU were mostly signs of infection and then one case each of injuries, hematuria, congenital anomalies, and anemia. The average hospital stay for neonates in both groups was four days (Table 4). 

**Table 4:** Neonatal outcome The data are presented as numbers, means, and percentages. The significance threshold was set at p < 0.05. NICU: neonatal intensive care unit; SCBU: special care baby unit; NC: not able to calculate due to a low number of cases.

Group	E (N = 208)	R (N = 39)	p-value
FHR abnormalities during labor N (%)	8 (3.84)	3 (7.69)	0.285
Neonates with complications after birth N (%)	59 (28.3)	13 (32.5)	0.598
Immediate complications			
Respiratory distress N (%)	1 (0.5)	0	NC
Perinatal asphyxia N (%)	4 (1.9)	0	NC
Early complications			
Infection N (%)	47 (22.6)	8 (20.5)	0.774
Hyperbilirubinemia N (%)	10 (5.1)	2 (4.8)	0.932
Shoulder dystocia N (%)	7 (3.3)	1 (2.5)	NC
Admission to NICU N (%)	5 (2.4)	0	NC
Admission to SCBU N (%)	15 (7.2)	1 (2.5)	0.279
Average hospital stay (mean ± SD)	4.2 ± 1.58	4 ± 1.47	0.557

Logistic regression analysis of neonatal outcomes revealed that a statistically significant number of neonates born via CD had more complications after birth, regardless of the type of analgesia that was used during labor (Table 5).

**Table 5:** Logistic regression analysis of neonatal complications OR: Odds ratio; SE: Standard error; z: z score; CI: Confidence interval; CD: Cesarean delivery. The significance threshold was set at p < 0.05.

Association with Neonatal Complications	OR	SE	z	p-value	95% CI
CD	2.84	1.12	2.63	0.008	[1.30-6.18]
Maternal analgesia with remifentanil	0.77	.29	-0.67	0.501	[0.36-1.64]
Maternal diseases	0.68	.28	-0.92	0.359	[0.30-1.53]

## Discussion

We evaluated the effects of EA and RA on neonatal outcomes. Parameters observed were immediate and early neonatal complications in mothers who received either EA or RA. We did not find any differences between groups regarding these complications. We measured an increased rate of complications in neonates born via CD. This finding is probably independent of the analgesia used during labor. Future studies should have a larger sample size and be powered to detect such associations.

Immediate fetal and neonatal complications in the first 24 hours after delivery that we registered through medical histories were FHR oscillations, respiratory distress, and perinatal asphyxia. Fetal bradycardia occurred in both groups, with a slightly more frequent occurrence in the R group, statistically nonsignificant. Although bradycardia is a known side effect of remifentanil in adult patients, many studies have confirmed this is not a typical occurrence in fetuses during labor [10]. Respiratory distress and perinatal asphyxia were the main reasons for neonatal admission to the NICU. All of these neonates admitted to the NICU belonged to group E. Although this does not indicate statistical significance between groups, a sample of larger size is required for its evaluation. Early neonatal complications occurred after the first 24 hours until discharge from the hospital. The most common complications were infections, and among these early neonatal complications, no difference was noticed between the groups.

The answer to complications observed in neonates could be found by analyzing the course of labor. The available obstetric outcomes registered through medical records in this research were the length of labor and delivery type. Both groups were found to be comparable with regard to these parameters. Prolonged labor is usually one of the indications for an ID or CD. As no group differences were found in the duration of labor, the delivery type was the same in both groups. This result is not consistent with previous studies [11], where it is very evident that EA prolongs the second phase of labor compared to remifentanil, thus complicating the course of labor and delivery type. Prolonging labor with analgesia used during the delivery certainly has a negative impact on neonatal outcomes [12]. The lack of differences in labor progress between the groups may be explained by the analgesia protocols employed in our hospital. In our hospital, we are initiating EA with cervical dilation of at least 4 cm after assessment by the responsible obstetrician. Previous studies have demonstrated that starting EA before a cervical dilatation of 4 cm was linked to a slower progression of labor [13]. However, many afterward studies performed did not confirm this claim [14-17]. Considering the retrospective nature of our research, we don't have enough evidence to support this conclusion. Another practice in our hospital that could affect the course of labor is a practice to wait for the urge to push (and even to stop the epidural to increase sensation). This is still practiced in some hospitals in Western countries [18]. This, in combination with the late initiation of EA, could be an explanation for the lack of influence of EA on labor progression in the present research.

When it comes to remifentanil and its effect on labor progression and neonatal outcome, some positive features make it very reliable for use in labor. Remifentanil is an opioid degraded by a nonspecific cholinesterase with a small distribution volume, a short onset time, and a plasma time-dependent half-life of three to five minutes. It is quickly eliminated following withdrawal from the drug [19,20]. No accumulation occurs following a long-term infusion, and the administration timing is unrestricted [21]. The pharmacokinetics of this short-acting opioid make it an ideal choice in labor with minimal impact on the neonate. Several meta-analyses have indicated that analgesia with remifentanil compared with EA resulted in shorter labor and a lower incidence of CD and ID [22,23]. Also, the incidence of maternal hyperthermia was lower. An adverse effect of remifentanil was maternal respiratory depression and episodes of desaturation, so this type of analgesia still requires caution, precise dosing, use of oxygen, and pulse oximetry [11,22,23]. Despite this, no worse neonatal outcome was found in the studies, although only parameters in the immediate period after delivery (Apgar score, perinatal asphyxia) were monitored.

Salameh et al., in their retrospective study on a sample of 2360 nulliparous women, monitored neonatal complications during the first 24 hours after delivery. They concluded that EA increases the chances of NICU admission, antibiotic use, neonatal birth trauma, positive pressure ventilation at birth, and respiratory distress on the first day of life. However, mothers with EA also had a longer second phase of labor, a higher rate of ID, meconium in amniotic fluid, fetal distress, and fever [9].

In their systematic review of 22 papers, Maroni et al. evaluated the effectiveness and safety of remifentanil in premature and term newborns. They examined the use of remifentanil for labor or cesarean section, neonatal procedural analgesia, and long-term sedation or analgesia in neonates. Neonates showed good adaptation after being exposed to remifentanil in the fetal period. This review found that remifentanil seems to be safe and effective in preterm and full-term neonates, but its safety is reduced by the possibility of chest wall rigidity [24].

Comparing EA and remifentanil during labor, as per the previous studies, it could be concluded that each method of analgesia during childbirth has its advantages and disadvantages [4-6]. EA could possibly affect the course of labor and have repercussions on the neonate in the period following delivery, while remifentanil has unfavorable sedation effects and causes desaturation in mothers, but due to its favorable pharmacokinetic properties, it has a lower tendency to affect the course of delivery and the condition of the neonate after delivery. In the current study, no differences were found in the outcome of neonates between these two groups of mothers. It is possible that modifying the administration of analgesia throughout labor may have the impact of reducing the impact of analgesia on labor and neonates.

The study has certain flaws and weaknesses. The retrospective nature of the research reduces the quality of the results. The conditions for performing analgesia were not controlled. Not all information was available through the information system, which could have affected the results. The sample of patients who received RA is small. Absence of a control group of subjects who delivered without analgesia.

## Conclusions

Neonatal outcomes and general labor characteristics, such as duration and delivery type, were not different between groups. A potential explanation for this conclusion could be the initiation of late epidural and the practice of waiting for the urge to push while slowing or stopping the epidural infusion. Neonates born via CD had an increased rate of complications. Further studies involving larger samples should be performed to confirm or reject the findings from this retrospective analysis.
